# University of the Pacific

**Published:** 1859-05

**Authors:** 


UNIVERSITY OF THE PACIFIC.—We learn from a circular that a medical college has been organized in San Francisco, with the following corps of professors: T. Morrison, M.D., Pathology and Practice of Medicine; Isaac Rowell, M.D., Chemistry; R. Beverly Coley, M.D., Physiology, Obstetrics, and Diseases of Women and Children; E. S. Cooper, M.D., Anatomy and Surgery; B. R. Carman, M.D., Materia Medica; and Hon. George Barston, Forensic Medicine.

The course of lectures will commence on the first Monday in May, and continue four months. We wish the faculty every success in their new enterprise, and think the establishment of a medical school in California must eventually be crowned with success.

